# Evaluation of Objective Uncertainty in the Visual System

**DOI:** 10.1371/journal.pcbi.1000504

**Published:** 2009-09-11

**Authors:** Simon Barthelmé, Pascal Mamassian

**Affiliations:** Laboratoire Psychologie de la Perception, CNRS, Université Paris Descartes, Paris, France; Northwestern University, United States of America

## Abstract

The role of sensory systems is to provide an organism with information about its environment. Because sensory information is noisy and insufficient to uniquely determine the environment, natural perceptual systems have to cope with systematic uncertainty. The extent of that uncertainty is often crucial to the organism: for instance, in judging the potential threat in a stimulus. Inducing uncertainty by using visual noise, we had human observers perform a task where they could improve their performance by choosing the less uncertain among pairs of visual stimuli. Results show that observers had access to a reliable measure of visual uncertainty in their decision-making, showing that subjective uncertainty in this case is connected to objective uncertainty. Based on a Bayesian model of the task, we discuss plausible computational schemes for that ability.

## Author Summary

Most work in vision science focuses on the question of why we perceive what we do, and we now have many models explaining what physical properties of a stimulus make us see depth, colour, etc. Here we ask instead what makes us feel *confident* in our visual perception: in the context of a visual task, what are the physical properties of the stimulus that will make us think we are doing the task well? The mathematical framework of Bayesian statistics provides an elegant way to frame the problem, by assuming that the visual system is trying to estimate physical properties of the world from incomplete, sometimes unreliable visual information. *Objective* uncertainty will therefore depend on the quality of the information available in the stimulus. In our experiments we compare objective uncertainty—as computed using the Bayesian framework—with subjective uncertainty, the confidence observers report about their visual percepts. To this end, we use a visual task with well-defined statistical properties, discrimination under noise. We report a surprising degree of agreement between objective and subjective uncertainty, and discuss possible computational models that could explain this ability of the visual system.

## Introduction

Every single human action happens in a context of uncertainty, being based on incomplete knowledge and undertaken despite unpredictable consequences. When faced with uncertainty, humans employ heuristics [1],[2] and show characteristic biases in their decision [3]. The neural structures involved in some of these decisions are now being identified [4]–[6]. Before one can make decisions that depend on uncertain information, the degree of uncertainty must be evaluated. The basic question of how well humans do at evaluating their own uncertainty remains largely understudied.

Uncertainty is a familiar concept in cognitive science, in particular thanks to Signal Detection Theory (SDT; Green and Swets 1966). In a typical psychophysical task, an observer has to detect small contrast increments near threshold. The uncertainty in this task comes mostly from internal variability: because of fluctuations in her internal representation of contrast, the observer makes mistakes and is uncertain about the correctness of her decisions. Unfortunately for the experimenter, this source of the uncertainty is internal to the observer and therefore only indirectly controllable.

Now consider another difficult perceptual task: listening to a speaker among cocktail-party chatter. Here the difficulty depends not so much on variability in the brain, but rather on interactions between the different voice signals: the one emitted by the speaker you aim to listen to, and the sound of other voices. Even with the volume of the other voices staying the same over time, difficulty will depend on the languages spoken, the gender of the speakers, and other sources of confusion. More generally, background chatter plays the role of noise, and difficulty will vary based on how much signal and noise covary.

An analogous visual task can be obtained by adding visual noise to a signal –random perturbations to the stimuli shown to the observer. Using visual noise, we are in a position to manipulate the *objective uncertainty*: objective uncertainty is inversely related to the amount of task-relevant information available in the stimulus. Concurrently, we can measure the *perceived uncertainty* of the observer, the level of confidence she actually reports. We introduce three experiments where we manipulate objective uncertainty and study its relationship with perceived uncertainty.

In the first two experiments, observers were presented with pairs of images of oriented objects embedded in high levels of noise, and had to report the orientation of the image of their choice. Even though the two images contained the same level of noise, the particular noise structure made one image orientation more certain than the other. We found that observers reliably chose the more certain of the two images, thereby providing evidence of a capacity to accurately evaluate objective uncertainty. We confirmed this in another experiment, in which we held the objective uncertainty of one of two stimuli fixed while varying the other, and asked observers to pick the less uncertain one. The greater the difference in uncertainty was, the greater the chance that observers picked the less uncertain stimulus, showing that uncertainty discrimination behaves similarly to normal psychophysical tasks. In a third experiment, we extend our results to a letter discrimination task. We discuss plausible computational mechanisms for achieving these results.

## Results

**Figure 1:**
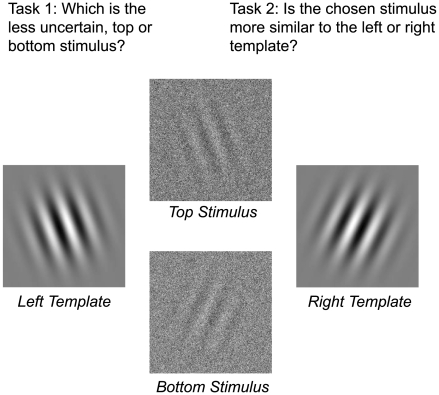
Layout of the experiment. The two templates appeared on the left- and right-hand sides of the screen. Two test stimuli were displayed simultaneously: they were computed from one of the two templates, to which noise was added. The two test stimuli had equal contrast, as illustrated here. Observers selected first which stimuli they felt more confident making an orientation judgment for (task 1). They were then asked to make that judgment (task 2).

In the first two experiments, visual uncertainty was introduced in an orientation discrimination task by manipulating the amount of pixel noise added to a visual template. There were only two templates, which were always visible to the observers. The templates were left and right oriented Gabor patches that presented alternating dark and bright lines under a blurry circular aperture (Figure 1). We embedded the templates in noise by adding a random perturbation to the luminance value of each pixel of the image, independently of the other pixels: the higher the variance of the random perturbation, the more noise. For high noise levels, one template can be mistaken for the other.

A decision must be reached by evaluating which of the two templates is the more probable hypothesis given the noisy stimulus. The orientation task can be understood as a classification task under noise, where stimuli correspond to items and the two templates determine the two categories: our two categories are simply defined as “stimuli generated by the left-tilted template”, and “stimuli generated by the right-tilted template”. An ideal Bayesian observer can be derived for this task, and we therefore defined the objective uncertainty of a stimulus as the entropy of the ideal observer's posterior distribution over the two classes.

Stimuli and templates can be represented as vectors in a space where dimensions correspond to the contrast of each pixel (difference to background luminance). Let **s** be the stimulus, **u** and **v** the templates (we use boldface notation for vectors). We assume that the characteristics of the noise are known and that the prior probabilities of the templates are equal. The posterior probability of template **u** is written:
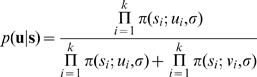
(1)Here *i* indexes the pixels from 1 to the total number *k*, and 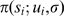 is the probability of observing value *s_i_* for a Gaussian of mean *u_i_* and standard deviation *σ.*

An ideal observer in the discrimination task will respond by choosing the most probable template. That decision function can be written using the log-likelihood ratio
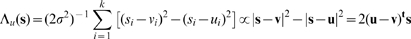
(2)The latter equality comes from the fact that 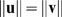, i.e., the templates have equal energy. The decision function simplifies to 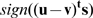.

What is uncertainty for our ideal observer? A very general measure of uncertainty is given by the information entropy of a probability distribution representing a state of knowledge [7]. Here the posterior distribution is binomial so the entropy can be written as
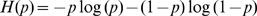
(3)When the natural logarithm is used, the entropy is measured in natural bits or nats [8] (since some of the entropy values in experiment 1 were very small, we used the log-entropy for computational convenience).We show in Text S1 that the entropy is monotonically related to the magnitude of the decision variable defined above, and that it corresponds geometrically to Euclidean distance to the decision boundary. Discrimination in white noise therefore provides a visual task in which objective uncertainty can be easily measured and manipulated, and compared to perceived uncertainty.

To measure perceived uncertainty we used comparative judgements. On every trial, observers saw a pair of noisy stimuli, one at the top of the screen, and one at the bottom. Of the two stimuli presented, they only had to make a discrimination judgement about one. In this setup, if observers want to maximize their discrimination performance, the best strategy is to choose the more certain of the two stimuli. This is precisely what observers were instructed to do: the task consisted in choosing, first, the stimulus for which they felt the more confident, and only then to make a discrimination judgment on the chosen stimulus (Figure 1). Note that choosing the better of two stimuli is independent of determining their nature (what template they are generated from). In the neurological condition of blindsight [9], patients are able to discriminate the visual properties of stimuli in a forced choice task but they largely underestimate their performance in this task. Observers' performance in the choice task will thus be a measure of their ability to access the objective uncertainty of each stimulus and to appropriately compare these uncertainties.

### Experiment 1

To determine whether observers did effectively pick the less uncertain stimuli, we contrasted two conditions. In the so-called True Choice (TC) condition, the two stimuli presented resulted from independent draws from the same noise distribution. Note that two stimuli with the same average noise level, as is the case here, can still vary in the objective uncertainty they induce, because different realizations of the same noise distribution can make the stimulus more or less ambiguous. In that case there is a benefit to be had in choosing the less uncertain of the two: this gives observers a higher chance of responding correctly than if only one stimulus is available.

**Figure 2:**
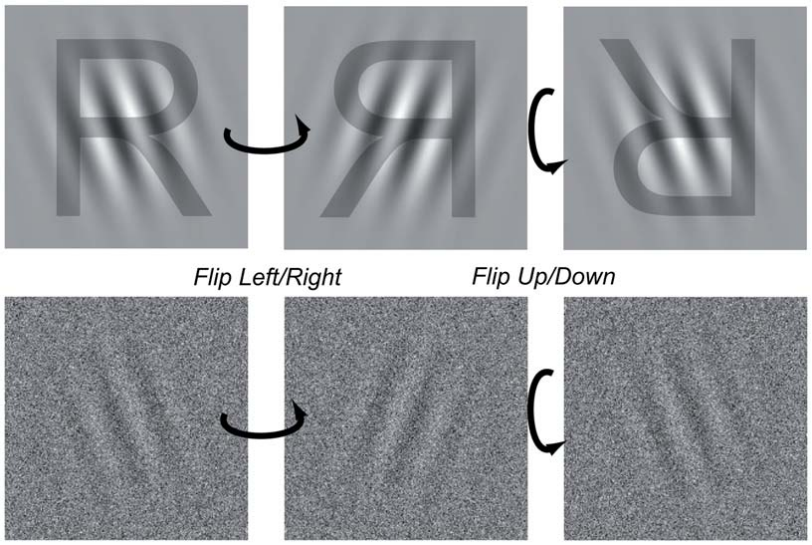
False Choice stimuli. The left-tilted can be flipped left-to-right to yield the right-tilted template. Another flip, this time up-down, yields back the left-tilted template. In the False Choice condition, we generated one of the stimuli at random, and used a left/right flip or a left/right flip followed by an up/down flip to produce a stimulus with equal uncertainty but different visual aspect. We superpose the shape of a R on the images to illustrate the transformations.

In the other condition, the False Choice (FC) condition, we removed that benefit: the first stimulus was computed the normal way, but the second was obtained by flipping the top one either once or twice (Figure 2). We took advantage of the underlying symmetry of our templates: flipping the first template left-to-right yields the second, and flipping the second bottom-top yields back the first. By applying these transformations to a noisy version of our template, we were able to create two stimuli that differed pixel-to-pixel, but were equivalent from the point of view of the classification task and thus carried *equal objective uncertainty* in that context. In the False Choice case, there is therefore nothing to be gained by choosing one rather than the other.

At no point in the experiment were observers aware of the existence of the two conditions. The two stimuli presented always had equal contrast, preventing observers from using a heuristic of selecting the lower-contrast stimulus as the most certain. The False Choice condition therefore provides the performance baseline that will be used to determine whether or not observers are able to successfully compare objective uncertainties.

We measured observers' performance, defined as proportion of correct classifications, in the two conditions across five different signal-to-noise ratios, chosen to span a range of performance between approximately 60 to 85%. Both the signal-to-noise ratio and the condition each trial belonged to were randomized. If observers are able to make accurate judgments of objective uncertainty, then we expect that measured performance will be higher in the TC than in the FC condition.

**Figure 3:**
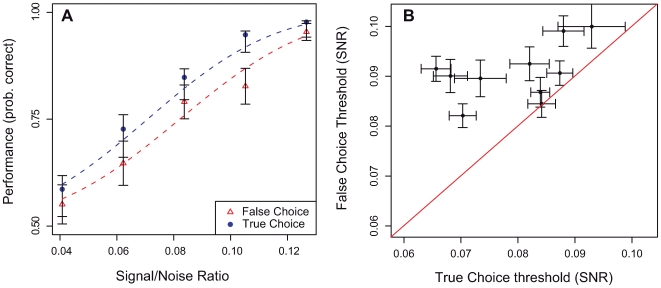
Results - performance. (a). Results for one observer. Each point represents measured discrimination performance (probability correct) for a given signal-to-noise ratio and condition. Two psychometric functions, one per condition, are fitted to measure performance. The psychometric functions are distinct, indicating that performance was higher in the TC condition. (b). Aggregated results. 75% thresholds are estimated from performance data separately for the two conditions. A higher threshold is indicative of lower performance. Error bars are standard errors obtained from a parametric bootstrap [32].

As expected given the nature of the task, mean performance for all observers grew with increased signal-to-noise ratio. More interestingly, however, mean performance is higher in the TC condition than in the FC condition, which translates into lower performance thresholds in the TC condition (Figure 3 a and b). To establish that the effect is genuine we used a model comparison technique. We used a likelihood-ratio test to evaluate the effect of True Choice versus False Choice (details in Text S1). Using two psychometric functions, one per condition, rather than one psychometric function for both conditions provides a significantly better fit to performance data (Nested hypotheses test [10]: p = 0.0004, 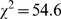, d.f. = 24).

It appears then that observers were able to take advantage of the True Choice condition, by choosing the less uncertain stimulus a majority of the time. It seems reasonable that, should the ability to pick the less uncertain stimulus be present, the probability of choosing the correct stimulus ought to be an increasing function of the magnitude of the difference: the more the two stimuli differ in their uncertainty, the more likely observers are to choose the right one. We evaluate that by regressing observers' choices of stimuli on the difference of log-entropies (Text S1). We found a highly significant effect (details in Text S1) of the difference in uncertainty on the probability of choosing the bottom stimulus: in other words, the more uncertain the bottom stimulus compared to the top one, the less likely observers were to choose the bottom one.

### Experiment 2

This last result hints at a more general property: in all psychophysical discrimination tasks, the larger the difference between two stimuli, the more reliable discrimination is. For example, when asked to compare the length of two lines, an observer's responses are likely to be better predictable when the two lines differ by 20 cm rather than 1. In a second experiment, we sought to confirm our findings by checking that discrimination of uncertainty behaves in the same way. The task was identical to that of experiment 1, but instead of introducing a False Choice condition, we manipulated the stimuli such that one – the standard – had always the same level of uncertainty and the other – the test – had lower uncertainty.

We show in the supplementary material that generating random stimuli with a controlled level of uncertainty can be achieved using a simple orthogonal projection. Mathematically, the space of all possible stimuli of the kind used here can be described in terms of the contrast of individual pixels by having one dimension (one axis) for each pixel. Then the two templates are two points **u**,**v** in that space, and stimuli obtained by adding white noise to a template are other points, forming Gaussian point clouds around the templates. To decide whether a point is more likely to belong to the left-tilted template rather than the right-tilted one, a simple geometrical rule describes the ideal strategy.

**Figure 4:**
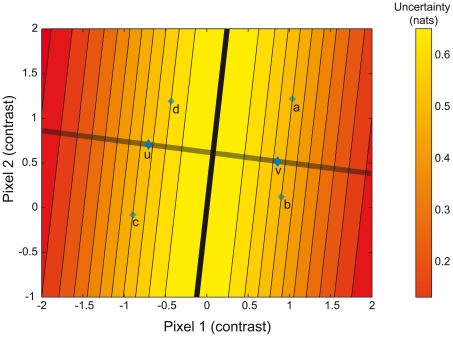
The orientation discrimination problem in stimulus space. The templates **u** and **v** are points in a space with dimensions corresponding to pixel luminances. Here we depict the problem for two pixels only. The optimal decision boundary – a plane - is represented by the blue line. Stimuli are obtained by starting from one of the two templates and adding a noise vector. They correspond to points in the space lying around **u** and **v**. The response is determined by which side of the plane they fall on. The closer they are from the decision boundary, the higher the chance that they could have been generated equally well from either template, and therefore the higher the uncertainty. Here, A,B and C are all points of equal uncertainty, whereas D has higher uncertainty. The uncertainty is given by the entropy of the posterior distribution, see Methods.

Imagine drawing a line between **u** and **v**, as in figure 4, where we illustrate the problem for stimuli with only 2 pixels. Now draw the plane (in higher dimensions; the hyperplane) that is orthogonal to the line and cuts through it at the mid-point. Then any stimuli falling on the same side of the plane as **u** we will call “left-tilted” and any falling on the side of **v** we will call “right-tilted”: the plane represents the decision boundary. Stimuli falling right on the hyperplane are completely ambiguous: both categories are equally likely. In fact, it is possible to show that the uncertainty of a stimulus is given by its (unsigned) distance to the decision boundary. Then the set of stimuli of fixed uncertainty is the set of points that are of the same distance to the decision boundary, and that set is simply the union of two parallel planes.

We therefore generated our stimuli by constraining them to lie on a plane of distance *d* to the decision boundary. Standard stimuli were always on a plane of distance *d_standard_* and test simuli were on a plane of distance *d_test_*. The difference between *d_standard_* and *d_test_* was varied parametrically between 4 different levels: we expected the observers to more reliably choose the test stimulus as the difference increased.

**Figure 5:**
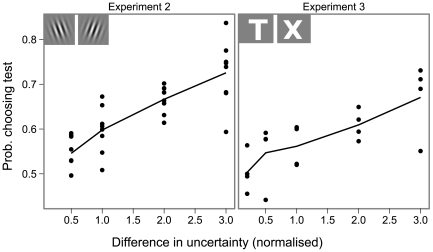
Results of experiment 2 and 3. In these two experiments, the test and the standard stimuli varied in uncertainty. We plot the proportion of times the test stimulus was chosen as a function of the difference between the uncertainty of the standard and the uncertainty of the test. The dots represent individual results, the solid line is the average over observers. Experiment 2 used orientation discrimination, experiment 3 used letter discrimination. The templates are shown on top of each graph. The levels of uncertainty are standardised across observers, see Text S1.

The results appear in figure 5: the larger the difference in uncertainty between standard and test, the more likely observers were to choose the test stimulus. We adapted the noise level to each observer's performance, so the distances used varied between observers. We normalise them with respect to the expected distribution of the distance to the hyperplane for the noise level chosen (see Text S1). The effect of the difference is significant for every observer as modeled by logistic regression of stimulus choice on difference in uncertainty (*t*-test for Generalised Linear Models coefficients, all p-values at 10^−3^ or below). This confirms that uncertainty behaves in that respect just like other psychophysical quantities: the more dissimilar two stimuli are on that scale, the more predictable observers' judgments are.

### Experiment 3

In experiments 1 and 2, the underlying visual task is orientation discrimination under noise, with templates identical in every way except for one basic attribute – their orientation. To check that our results were sufficiently general, we ran a variant of experiment 2 using a letter discrimination task. Observers had to discriminate between the letters ‘T’ and ‘X’ (shown on figure 5), a pair chosen because the corresponding characters correlate very little. Except for the nature of the templates, experiment 3 was identical to experiment 2 and we replicated its results (figure 5): observers were more likely to pick the less uncertain stimulus when the difference in uncertainty was larger. Our results thus generalize to more sophisticated visual tasks.

### Computational models

Our results imply that observers had access to some estimate of the uncertainty in the orientation task. How is that estimate computed? Do observers have effective access to a probability distribution over perceptual hypotheses, from which they can estimate their own uncertainty? Or do they rely on more limited information? To investigate that question we evaluated two distinct families of models that compute uncertainties globally over the full distribution for the first, and locally for the second.

We begin by defining the following quantities: let **r** and **s** be two stimuli, represented as vectors of pixel luminances. Call **u** and **v** the left-tilted and right-tilted templates. Then 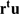 and 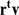 are measures of how “different” **r** is to **u** and **v**, respectively. If **r** is more like **u** than **v** (i.e., 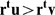), then it is more likely to have been generated from **u**, and hence the observer should respond “left-tilted” for stimulus **r**.

In comparing the uncertainty between two stimuli - choosing between **r** and **s** - the following procedure is exactly equivalent to the strategy of the “ideal observer” (i.e., the strategy that maximizes performance, see Text S1). Compute 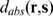 as
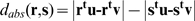
(4)and choose **r** if 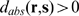, **r** otherwise. This corresponds to evaluating uncertainty based on the full posterior distribution (see equation 1): uncertainty is low if one hypothesis corresponds to the data much better than the other, and high otherwise. We call this model the *difference of responses* model.

Another strategy, perhaps simpler for the observer, is to evaluate uncertainty based only on how well the best hypothesis fits the data. We call this the *maximum response* model. The same measures of distances are computed as in the first model, but only the maximum is retained for each stimulus. The observer then compares the two maxima
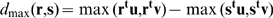
(5)Put into perceptual terms, this corresponds to a strategy of picking the stimulus that seems to have a more salient dominant orientation, when the templates were Gabor patches, or the stimulus that was more “letter-like”, when the templates were characters. In statistical terms this is equivalent to evaluating uncertainty based on the magnitude of the likelihood of the maximum-likelihood hypothesis (Methods), a strategy that is sub-optimal for our task but still gives an improvement over choosing between the two stimuli at random.

Both hypotheses are realistic from a neural-computation point of view. Computing 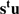 and 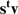 is nothing more than a linear filtering of the neural input: although some important non-linearities have been identified in visual orientation discrimination, linear filtering remains the basic operation in all models [11],[12]. Computing the decision variables, whether *d_abs_* and *d_max_*, is a simple non-linear step readily implementable in a neural system.

To test those models we make the same assumption we did for regressing choice on difference in log-entropy: the higher *d_abs_* and *d_max_*, the more likely observers are to choose the bottom stimulus. As above, we compute the decision variables for every trial and we fit a linear binomial regression model to the responses (Text S1).

**Figure 6:**
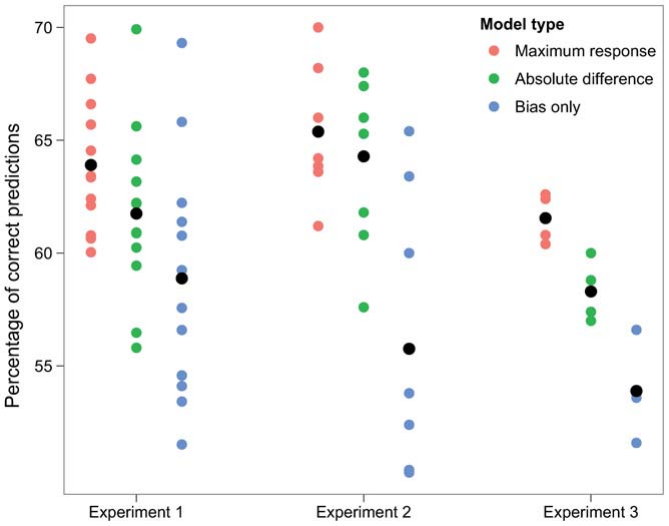
Proportion of correct predictions for three models of choice. The maximum of response and absolute difference models are presented in the text. Observers presented a bias in their choice of stimuli (most of them choosing the top one with a proportion higher than chance), so we plot the proportion correct of a model that predicts observers always choosing the stimulus to which they are biased (Bias only, see Methods).

Our models give for each trial a choice probability. On figure 6 we plot the percentage prediction correct (i.e., the proportion of trials where the model predicted with p>.5 the choice the observer actually made). The two models have the same number of degrees of freedom, and can be directly compared. Both predict the data significantly better than chance, but the maximum response has a significant lead. Our data therefore point to a likelihood-based evaluation of visual uncertainty, rather than one based on the full posterior distribution.

## Discussion

In summary, we demonstrate here that humans display *second-degree* knowledge of a visual discrimination task: not only are they able to detect what signal is in the noise (first-degree knowledge), but also to estimate how uncertain that knowledge is, at least comparatively. Why humans should be so well calibrated to what is in essence a laboratory task rather than a natural one is a question that deserves attention. It is possible that they learn the statistical properties of the task over time, although we find no conclusive evidence for that in our data (see Text S1).

Previous research lacked an objective standard to compare subjective judgements to, and relied on ratings [13]. Various biases have been reported in human confidence judgments, including over- and under-confidence, global/local inconsistencies, as well as inter-cultural differences [14]–[17]. The forced-choice method we outlined here allows one to test human observers' objective capacity to detect differences in uncertainty contained in a task, and to evaluate possible computational mechanisms much more rigorously. It is a potentially important methodology in the study of discrepancies between visual performance and confidence, a topic many believe to be connected to the wider issue of awareness [18],[19], but potentially also in investigations of metacognition in non-human species [20],[21].

Our work is in tune with a variety of current research that tries to understand visual function as a form of Bayesian inference [22]–[25]. These theories posit that the visual system explicitly encodes probability distributions over perceptual hypotheses. In that context, it makes intuitive sense that the system should be able to measure the uncertainty of such a distribution: comparing two uncertainties as we do here is rarely needed as such, but comes into play in more complicated decisions. Just as a low feeling of confidence in an item to be memorized is a clue that further study is needed [26], high visual uncertainty signals that more information is needed, making precise evaluation of visual uncertainty an essential aspect of exploration mechanisms [27].

The results given here agree with other studies that have found unexpectedly accurate decision-making in perceptual [28],[29] and motor systems [30],[31]. These results imply that uncertainty is dealt with at an implicit level: unlike them, we require observers to make explicit comparisons between levels of uncertainty. The observers who took part in our experiment nevertheless found the task quite intuitive: indeed, we often make comparative judgments of visual uncertainty “in the wild”, as when we judge if we see better from one vantage point than another.

Generally, we expect that confidence measures have the potential to play a larger role in computational investigations of perceptual decision-making. The evaluation of uncertainty is a necessary first step in any statistical decision-making system, and biases and approximations in evaluating uncertainty will cause sub-optimal decisions. A systematic study of the evaluation of uncertainty in the visual system will help uncover the shortcuts taken by the brain in making perceptual decisions.

Our method can be generalized to other noise models, other sensory modalities, and other tasks. But showing that fine-grained discrimination of uncertainty can be done is of course not an end in itself: uncovering how that essential operation is achieved in the brain is a natural next step.

## Methods

Additional and more complete methods can be found in Text S1.

### Ethics statement

This study was conducted according to French guidelines on research involving human participants. All participants gave informed consent.

### Experiment 1

#### Stimuli

The templates used were Gabor patches, with a standard deviation of 1.4. Observers viewed the stimuli from a distance of 57 cm. Uncorrelated (white) Gaussian noise was added to the templates to produce the stimuli.

#### Observers

12 observers took part in the first experiment. All observers had normal or corrected-to-normal vision and gave informed consent.

#### Experimental setup

*General*. Observers were familiarised with the task with a 20-trial run of the experiment, during which the experimenter was present. They completed a total of 1000 trials over the course of two sessions. We varied the signal-to-noise ratio of the stimuli randomly, trial by trial. Feedback on the orientation task was provided on every trial.

*False Choice and True Choice conditions*. On each trial, a condition was chosen pseudo-randomly. In the True Choice condition, the two stimuli were generated independently from the same noise distribution. This was done to ensure that the two images had equal contrast, and that observers could not use that clue to discriminate between less certain and more certain stimuli. In the False Choice condition, the first stimulus was computed as in the True Choice condition, but the second was obtained by flipping the first either left-to-right or left-to-right followed by up-down. This made it possible to have two stimuli that were different pixel-to-pixel, and looked different to the observer, but contained the same amount of information (i.e., had the same entropy).

### Experiment 2

The experimental method was the same as in experiment one, unless indicated otherwise.

#### Stimuli

The templates used were Gabor patches, presented in a square window subtending 5 degrees of visual angle. The stimuli were generated by adding uncorrelated noise, then projecting onto an equal-uncertainty hyperplane as described in Text S1.

#### Observers

8 observers took part in the second experiment, including the first author. All observers had normal or corrected-to-normal vision and gave informed consent.

#### Experimental setup

Observers were familiarised with the task with a 10-trial run of the experiment, during which the experimenter was present. Observers then completed a total of 500 trials in one session. The difference in uncertainty between the test and the standard stimuli was chosen at random on every trial, between four different levels (see Text S1). Feedback on the orientation task was provided on every trial.

### Experiment 3

The experimental method was the same as in experiment 2, unless indicated otherwise.

#### Stimuli

The templates used were a T and a X, rendered in a sans-serif font. The templates are shown on figure 5. The contrast of the templates was adjusted so that they had equal energy.

#### Observers

4 observers took part in the third experiment, including the first author. All observers had normal or corrected-to-normal vision and gave informed consent.

#### Experimental setup

Observers were familiarised with the task with a 10-trial run of the experiment, during which the experimenter was present. Observers then completed a total of 500 trials in one session. The difference in uncertainty between the test and the standard stimuli was chosen at random on every trial, between four different levels (see Text S1). Feedback on the orientation task was provided on every trial.

## Supporting Information

Text S1 Supporting Information.
(1.21 MB DOC)
