# Supplementary material for: Evaluation of Objective Uncertainty in the Visual System
Source: PLoS Comput Biol. 2009 Sep 11;5(9):e1000504. doi: 10.1371/journal.pcbi.1000504 (PMC2730538; doi:10.1371/journal.pcbi.1000504)
Supplement: Text S1 — Supporting Information. (1.21 MB DOC) [file pcbi.1000504.s001.doc]

Evaluation of Objective Uncertainty in the Visual System

Simon Barthelmé, Pascal Mamassian

Supporting information

PLoS Computational Biology

**1. Detailed experimental methods**

**Stimuli.** Observers viewed the stimuli from a distance of 57 cm. Stimuli were displayed on Sony GDM-C250 calibrated monitor, driven by an Apple G5 PowerPC running Matlab 7 (Mathworks, Inc.). The Psychtoolbox library [1,2] was used for stimulus control.

For the purposes of the categorization task, pseudo-random Gaussian noise was added to the stimuli. We represented stimuli as vectors of luminance values, with a value of 0 standing for the background luminance, and -1 and 1 standing for the lowest and highest luminance values achievable on the monitor, respectively. Stimuli were generated by summing the template and noise components

where **s** is the stimulus, **t** the template, and **n** the noise. The vector **n** had independent and identically distributed Gaussian elements with mean 0 and standard deviation σ.

To avoid clipping luminance values because of the limited range of computer monitors, we kept template contrast very low, so that low noise contrasts would be sufficient to induce less-than-perfect performance.

We defined the signal-to-noise ratio as , where *c* stands for the highest value of the template.

**Templates.** For the orientation discrimination task (experiments 1 and 2), we used two Gabor patch templates that had the same contrast, phase and frequency. They differed only in orientation, with one appearing “tilted to the right” (orientation: +20 degrees from vertical, clockwise), and the other “tilted to the left” (-20 degrees). For the letter discrimination task (experiment 3), we used a T and a X, rendered in a sans-serif font (Bitstream Vera Sans), slightly edited to equalise the number of white pixels. The templates are shown on figure 5 of the main text. The contrast of the templates was adjusted so that they had equal energy.

**Task**. The main task was discrimination under noise. On each trial, observers viewed two noisy stimuli that were obtained from either of two templates, with pixel noise added. They were instructed to choose the stimulus for which they felt the least uncertain (the one for which they felt they had the greater chance of making a correct discrimination judgement), and to make an discrimination judgment (orientation or character) for the stimulus they had picked. They responded by means of pressing the arrow keys on a standard keyboard. Both stimuli, as well as the two templates, were visible throughout the trial. The two stimuli were displayed above and below centre. The templates were displayed on the left- and right-hand sides of the display.

*Experiment 1*

Observers were familiarized with the task with a 20-trial run of the experiment, during which the experimenter was present. They completed a total of 1000 trials, over the course of two sessions. We varied the signal-to-noise ratio of the stimuli randomly on each trial. All observers were exposed to five different levels, chosen to span approximately the range of 60 to 85% correct. The values were determined prior to the experiment by running the observers through an adaptive version of the standard categorization task (see **Prior measurement of individual psychometric functions and threshold**).

*Experiment 2 and 3*

Observers were familiarized with the task with a 10-trial run of the experiment, during which the experimenter was present. They completed a total of 1000 trials, over the course of two sessions. We varied the difference in uncertainty between the standard and test stimuli, with a scale defined by the distribution of uncertainties for the observer’s threshold signal to noise ratio (see **Uncertainty levels in experiment 2 and 3**). The levels were 0.5, 1, 2 and 3 standard deviations, in experiment 2, and 0.2, 0.5, 1, 2 and 3 in experiment 3. The observers’ threshold were determined prior to the experiment by running the observers through an adaptive version of the standard categorization task (see **Prior measurement of individual psychometric functions and threshold**).

Feedback was given on the discrimination task at the end of every trial: the words “Correct” or “Incorrect” appeared on the screen.

**False Choice and True Choice conditions*.*** On each trial, a condition was chosen pseudo-randomly. In the True Choice condition, the two stimuli were generated independently from the same noise distribution. This was done to ensure that the two images had equal contrast, and that observers could not use that clue to discriminate between less uncertain and more uncertain stimuli.

In the False Choice condition, the first stimulus was computed as in the True Choice condition, but the second was obtained by flipping the first either left-to-right or left-to-right followed by up-down. This made it possible to have two stimuli that were different pixel-to-pixel, and looked different to the observer, but contained the same amount of information (i.e., had the same entropy).

We emphasize that observers could not discriminate based on total power in the image spectra around the spatial frequency of the two templates. What is relevant in the frequency domain is the difference in power between the respective orientations of the two templates. One could have high total power and little difference, indicating high uncertainty. We explore that issue further in the section on **Overall stimulus contrast and stimulus choice**, below.

**Prior measurement of psychometric functions and thresholds.**

*Experiment 1.* In order to obtain a rough estimation of observers' performance on orientation discrimination, prior to the main experiment, we asked observers to perform a classical orientation discrimination task. Only one stimulus was displayed, and observers had to discriminate its orientation. The same type of stimuli was used as in the main experiment. Observers completed 150 trials. The signal-to-noise ratio was varied according to an adaptive procedure that tried to maximize around the threshold of the psychometric function. A psychometric function was then estimated for each observer, and SNR levels in the subsequent main experiment were chosen accordingly.

*Experiment 2 and 3.* We noticed in earlier versions of the experiment that naïve observers occasionally had a surge in performance after the first few dozen trials, so we ran a short training session on the categorization task prior to the experiment to ensure that observers’ performance had stabilized. Observers ran first 60 “easy” trials at a high SNR, then 3 blocks of 75 trials. During those blocks the SNR was varied adaptively to measure the 70% threshold, using a Bayesian method we designed (a description of the method can be found on the first author’s website). The estimated threshold derived from the last block was kept as the final estimate as more representative of a stabilized performance level.

**2. Mathematical analysis**

**Relationship between entropy and log-likelihood ratio** In our case, comparing the uncertainty of two stimuli can be achieved by just considering the log-likelihood ratio, without computing the entropy. Here the log-likelihood ratio is equal to the log-ratio of posterior probabilities:

Taking the opposite of the absolute value, and treating the resulting quantity as a function of *p*(**u**|**s**), we obtain a concave function over (0,1) that has a maximum and is symmetrical around 0.5:

In other words (with the entropy of a Bernoulli distribution):

**Estimating stimulus uncertainty based on filter responses.** Three models were considered for the choice of stimuli, *bias only*, *difference of responses*, and *maximum of responses*. Here we examine the probabilistic basis for the latter two. We show in the main text (Methods) that implies and that the log-likelihood ratio is proportional to . Let **r**,**s** stand for the two stimuli presented to the observer on a given trial. Then,

This implies that choosing stimuli based on is equivalent to choosing the stimulus that has the least posterior uncertainty, the basis for our difference of responses model.

Consider now an observer that chooses stimuli according to the following strategy:

Choose **r**, the bottom stimulus, if:

We will show that this is implemented in the maximum response model described in the text.

We have:

therefore:

(is a function of σ only), and:

We know that **u** and **v** have the same energy, and in our case **r** and **s** have equal energy as well (the energy of the noise is the same): and .

We deduce (after some straightforward algebra):

That decision strategy evaluates the maximum likelihood hypothesis for each stimulus, and chooses the one that has the highest maximum likelihood value.

Computing **s^t^u** and **s^t^v** can be achieved via simple linear filtering of the visual data with filters based on the templates (realistically, non-ideal templates will be used). The only non-linear step involved in these models is the extraction of the decision variable, but the resulting functions are linear by part, thus not very complex to compute.

**Stimulus uncertainty and distance to the decision hyperplane.** Let . Then the decision boundary of the decision function is:

This defines a hyperplane in with normal vector **w**. The absolute log-likelihood ratio, and therefore the uncertainty, is proportional to . Therefore

is a level set of the uncertainty. It is easy to see that this is the union of two hyperplanes parallel to the decision boundary, and at the same distance to it (here distance between hyperplanes *H* and *H’* is defined as the distance between their two closest points).

The decision boundary is also the set of stimuli with maximal uncertainty.

**Figure S1**. Controlling stimulus uncertainty. The set of all stimuli with the same uncertainty is represented by two hyperplanes in stimulus space, shown here in gray. We generate stimuli by adding noise to one of the templates, then projecting on the closest hyperplane between the two. The resulting stimuli always have the same level of uncertainty.

**Constraining stimulus uncertainty**. Stimuli obtained by adding an independent noise component have a random level of uncertainty. In the second experiment we modified the method of generating stimuli so that they were as close to normal stimuli as possible, while their uncertainty could be manipulated as desired. The process is illustrated in figure S1.

We show above that the solution set of:

is a set of stimuli with equal uncertainty. The scalar *a* sets an uncertainty level. Starting from a stimulus **s**, obtained as in the first experiment through equation , we want to find the stimulus **x** such that **x** is as close as possible to **s** and its uncertainty level is *a*. This defines a constrained optimisation problem:

The solution is found by putting the problem in Lagrangian form and setting the derivatives to 0.

This corresponds to orthogonal projection on the hyperplane of uncertainty level *a*, which of course makes intuitive sense.

The resulting stimulus statistics can also be derived analytically:

Recall that **s = t + n,** with **n** an independent Gaussian noise vector and **t** equal to either **u** or **v**. From equation , we see that **x** is an affine function of **n,** therefore **x** is Gaussian as well and its mean and covariance are given by:

Generating stimuli using the constrained-uncertainty method by way of equation is therefore equivalent to adding non-white noise of mean and covariance

**Uncertainty levels in experiments 2 and 3**. For an unmanipulated stimulus **s = t + n** of the kind used in experiment 1, the mean decision variable is given by:

and its variance is given by:

We set the uncertainty of the standard stimulus to the value of (17). The test uncertainty was always less than the standard uncertainty, and we express it in terms of standard deviation .

**3. Data analysis**

**Psychometric functions.** The psychometric functions were modeled as cumulated Gaussian functions. Fitting such a psychometric function is equivalent to fitting a general linear model with a modified binomial link and binomial responses [3]. We used R’s glm facility for fitting and model comparison [4].

**Successful discrimination of uncertainties and performance***.* In experiment 1, to test the experimental hypothesis that observers did on average choose the less uncertain of two stimuli, we performed a likelihood ratio test based on the following models:

Null hypothesis. The null hypothesis predicts equal performance in the TC and FC conditions, across SNR levels. In psychophysical terms this is equivalent to one underlying psychometric function per observer that explains both sets of responses.

Experimental hypothesis. The experimental hypothesis predicts higher mean performance in the TC condition as opposed to FC. One would therefore need two psychometric functions to properly describe observers' performance.

This hypothesis can be tested statistically by fitting two models for each observer, using a maximum likelihood approach. One model, corresponding to the null hypothesis, has only two free parameters to describe performance as a function of SNR, for both the FC and the TC conditions. The alternative model has two free parameters per condition (2 times 2 for shape). The two models were fit using maximum-likelihood. The likelihood-ratio statistic:

where *L_EH_* and *L_NH_* are the maximized likelihoods of the experimental and the null hypotheses, has a distribution with degrees of freedom equal to the additional free parameters in the more complex model. Two observers out of twelve were excluded from the analysis because their measured performance was above 75% for all SNR levels and the psychometric fit was poorly constrained as a result.

**Predicting choices from evaluations of uncertainty***.*

For experiment 1, we used linear regression with binomial responses to predict observers’ choices of stimuli from differences in stimulus uncertainty. This is an instance of a Generalised Linear Model. We chose Cauchy link functions because they provide higher tolerance to outliers, which human observers are known to generate. The use of probit (cumulated Gaussian) or logit (logistic) links does not impact the results in a major way.

To evaluate the global effect of differences in uncertainty, as measured by the entropy of the posterior distribution, we regress aggregated responses for all trials to the difference in log-entropy:

 is the predicted probability of preferring the top stimulus to the bottom stimulus, with *i* indexing trials and *o* observers, and is a smooth inverse link function (here the Cauchy link). We allow two free parameters per observer, the weights *w*. The difference in log-entropy for trial *i* is given by .

The weight parameters are estimated using maximum likelihood. All of the *α* coefficients come out significant at the 0.05 level or below (testing for the effect of individual predictors in a GLM), and all are positive, indicating that observers are more likely to choose the bottom stimulus when it is less uncertain.

**Difference and max models**. For comparing the two models we describe in the text (“difference of responses”, “maximum response”) we fit a binomial linear model to the data, with two parameters per observers, using the same functional form as above. In addition, we fit a model that took no account of stimulus uncertainty, but only of observers’ biases in their choice of stimuli, the *Bias Only* model. That model has the form

where o runs over observers. It therefore predicts an identical probability for every choice a given observer makes.

Model performance can be compared based on deviance statistics (deviance can be likened to a residual sum of squares for generalized linear models) or on percentage correct. We compute percentage correct as:

Θ stands for the Heaviside step function, gives the model’s prediction for probability of choosing stimulus , and *c_i_* is the observed choice: *c_i_* = 1 if was chosen, 0 otherwise.

*Experiment 1.*

A likelihood-ratio test for nested models shows that the *maximum response* and the *absolute difference* models are superior to the *bias only* model (*Maximum response versus bias only* p < 0.01,= 405,d.f.=12 *Absolute difference versus bias only* p < 0.01,= 234.7,d.f.=12).

To compare *maximum response* and the *absolute difference* models we used standard bootstrap replicates of the difference in percentage correct and deviance (1000 replicates). The difference in percentage correct is in favor of the *maximum response* model (mean difference = 2%, s.d = 0.007%, one-tailed *t-*test p < .01) and so is the difference in deviance (mean difference = -172.42, s.d. = 31.86, p < .01).

*Experiment 2*

The analysis was replicated in the case of experiment 2 with identical results: the *maximum response* and the *absolute difference* models are superior to the *bias only* model (p < .01), and the *maximum response* model is superior to the *absolute difference* model in terms of deviance (mean difference in bootstrap replicates: -32.77, p < .01) and percentage prediction correct (mean difference in bootstrap replicates: 0.65%, p < 01).

**Figure S2**. Stimulus contrast as a function of stimulus uncertainty. 1000 stimuli were generated according to the noise distribution used in experiment 1, and their uncertainty and RMS contrast were measured.

**Overall stimulus contrast and stimulus choice.** In the two experiments, the difference in contrast between the two stimuli was independent of the difference in uncertainty (in other words, there was no point in choosing the stimulus with the less contrast).

To see this, refer to figure S2. We recreated a sample of a 1000 stimuli with the same characteristics as the ones used in our experiment, at a signal-to-noise ratio of 0.08 (typical of the values that occurred in experiment 1). We plot stimulus uncertainty, as measured by the ideal metric *d_abs_,* in relation to root-mean-square stimulus contrast. The complete lack of relation is clearly visible. The signal contrast varies very little (standard deviation of 5.40x10^-4^ ), and there is a complete absence of correlation between contrast and uncertainty.

We nevertheless ran a statistical analysis to check that the difference in contrast was not predictive of observers' choices. We report the results for the second experiment, the results for the first experiment are similar.

We first tested for a parametric effect using a generalised linear model that models probability of stimulus choice as a smooth, parametric function of difference in contrast – in the case of a logistic link, this is equivalent to running a logistic regression of stimulus choice on stimulus contrast. For the logistic, probit, and Cauchy links we found no significant effect of difference in contrast (two-tailed *t* test, p-value of 0.74 for all links).

The GLM assumes a parametric relationship, but we also checked for a potential non-parametric relationship using a Generalised Additive Model [5] with a smooth, unknown effect of difference in contrast and a logistic link. A test for the non-parametric effect gives a p-value of 0.51.


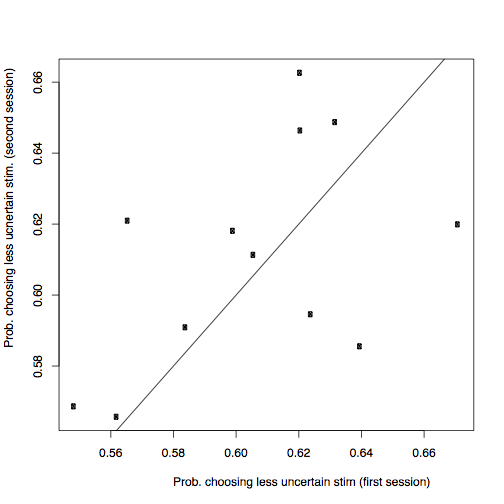


**Figure S3**. Probability of choosing the less uncertain stimulus in the first and second sessions of experiment 1. A learning effect would cause higher score in the second session than in the first. No significant effect was found (see text).

**Effects of learning**. Observers were given feedback on every trial, so it is possible that their capacity to discriminate uncertainties improved over time. We looked for evidence of such a learning process in the data for Experiment 1, where it was more likely to occur (unlike the others, experiment 1 took place over two sessions on different days). We take as a measure of performance in the evaluation of uncertainty the probability of choosing the less uncertain stimulus on a given True Choice trial. We separated the trials into those of the first and those of the second session, and computed the probability of a correct choice for each observer and each session (figure S3). We find no significant effect of learning: a GLM regression of the probability of a correct choice on the session gives a p-value of 0.675 (Cauchit link, z = -0.419). A regression on trial number gives a similar non-significant result.

References

1. Brainard DH (1997) The Psychophysics Toolbox. Spatial Vision 10: 433-436.

2. Pelli DG (1997) The VideoToolbox software for visual psychophysics: transforming numbers into movies. Spatial Vision 10: 437-442.

3. Yssaad-Fesselier R, Knoblauch K (2006) Modeling psychometric functions in R. Behavior Research Methods 38: 28-41.

4. R Development Core Team (2007) R: A Language and Environment for Statistical Computing. Vienna, Austria: R Foundation for Statistical Computing.

5. Hastie T, Tibshirani R (1990) Generalized Additive Models: Chapmann & Hall.
